# Hepatitis C Virus NS5A Protein Triggers Oxidative Stress by Inducing NADPH Oxidases 1 and 4 and Cytochrome P450 2E1

**DOI:** 10.1155/2016/8341937

**Published:** 2016-04-20

**Authors:** Olga A. Smirnova, Olga N. Ivanova, Birke Bartosch, Vladimir T. Valuev-Elliston, Furkat Mukhtarov, Sergey N. Kochetkov, Alexander V. Ivanov

**Affiliations:** ^1^Engelhardt Institute of Molecular Biology, Russian Academy of Sciences, Vavilov Street 32, Moscow 119991, Russia; ^2^CRCL, INSERM U1052, CNRS 5286, Université de Lyon, 151 Cours A. Thomas, 69424 Lyon Cedex, France

## Abstract

Replication of hepatitis C virus (HCV) is associated with the induction of oxidative stress, which is thought to play a major role in various liver pathologies associated with chronic hepatitis C. NS5A protein of the virus is one of the two key viral proteins that are known to trigger production of reactive oxygen species (ROS). To date it has been considered that NS5A induces oxidative stress by altering calcium homeostasis. Herein we show that NS5A-induced oxidative stress was only moderately inhibited by the intracellular calcium chelator BAPTA-AM and not at all inhibited by the drug that blocks the Ca^2+^ flux from ER to mitochondria. Furthermore, ROS production was not accompanied by induction of ER oxidoreductins (Ero1), H_2_O_2_-producing enzymes that are implicated in the regulation of calcium fluxes. Instead, we found that NS5A contributes to ROS production by activating expression of NADPH oxidases 1 and 4 as well as cytochrome P450 2E1. These effects were mediated by domain I of NS5A protein. NOX1 and NOX4 induction was mediated by enhanced production of transforming growth factor *β*1 (TGF*β*1). Thus, our data show that NS5A protein induces oxidative stress by several multistep mechanisms.

## 1. Introduction

Hepatitis C virus (HCV) is a blood-borne pathogen with ca. 120–170 million chronic carriers worldwide [1]. Chronic hepatitis C (CHC) infection is often accompanied by various liver and extrahepatic diseases that include fibrosis, steatosis, and insulin resistance and frequently progresses to cirrhosis and liver cancer [1]. Investigation of molecular mechanisms which underlie the pathogenicity of the virus led to the discovery of multiple events in CHC carriers that can trigger metabolic dysfunctions and on the long term carcinogenesis. In particular, studies from several groups pointed out oxidative stress as a phenomenon which is strongly associated with most of the HCV-associated diseases ([2–4] and references herein).

Oxidative stress is a marked increase of highly reactive oxygen intermediates (reactive oxygen species, ROS) including superoxide anion, hydroxyl radical, and hydrogen peroxide [5]. ROS readily modify various biological compounds including nucleic acids, proteins, and lipids, thus presenting a threat to cell fate. In cells, ROS are normally present at low levels and formed by various enzymes. These include the oxidative phosphorylation system in mitochondria, ion channels such as NADPH oxidases (NOX), metabolic enzymes such as xanthine oxidase, enzymes involved in degradation of lipids, biogenic polyamines and cytochromes, and finally members of the protein folding machinery at the endoplasmic reticulum such as the ER oxidoreductins (Ero1) [5, 6]. It has been shown by several groups that five of the HCV proteins, namely, core, NS5A, and to a lesser extent E1, E2, and NS4B [7–9], induce oxidative stress by two major mechanisms. These include alteration of calcium homeostasis leading to mitochondrial dysfunction [10, 11] and induction of NOX1 and NOX4 [12, 13].

To date it has been shown that core protein can induce efflux of Ca^2+^ from ER to mitochondria by several different mechanisms, alter normal functioning of mitochondria (summarized in [2]), and induce NOX4 enzyme in a transforming growth factor *β*1- (TGF*β*1-) dependent manner [13]. In addition, we have recently demonstrated that HCV core also induces NOX1, cytochrome P450 2E1 (CYP2E1), and ER oxidoreductin 1*α* (Ero1*α*), with the latter being a mediator of calcium perturbations and generation of superoxide in mitochondria [14]. In contrast to core protein, the mechanisms by which NS5A induces oxidative stress remain more obscure. The only data available show that similar to core protein, NS5A triggers passive leakage of calcium ions from the ER [9], an event that has been shown to be associated with elevation of ROS levels [15].

The goal of our study was to investigate the mechanisms by which NS5A induces ROS using as model Huh7 cells expressing NS5A protein. A particular focus was to be given to NADPH oxidases 1 and 4 and other ROS-generating cellular enzymes such as ER-residing cytochrome P450 2E1 and ER oxidoreductin 1*α*. In addition, we aimed to identify the domains of NS5A containing prooxidant activity.

## 2. Materials and Methods

### 2.1. Materials

Lipofectamine 2000 was from Invitrogen (Carlsbad, CA, USA), Dulbecco's modified Eagle medium (DMEM) and antibiotics for cell cultures were purchased from PanEco. Fetal bovine serum (cat #SV30160.03) was obtained from HyClone (Logan, UT, USA). 2′,7′-Dichlorodihydrofluorescein diacetate (H_2_DCFDA), dihydroethidium (DHE), and other reagents were from Sigma (St. Louis, MO, USA), unless otherwise noted. HRP-conjugated antibodies to c-myc tag (18824P) were from QED Bioscience (San Diego, CA, USA), primary antibodies to cytochrome P4502E1 (ab28146), *β*-actin (ab3280), HCV core (ab58713), and HRP-conjugated anti-rabbit and anti-mouse secondary antibodies, as well as CYP2E1 inhibitor 4-methylpyrazole were purchased from Abcam (Cambridge, UK). Antibodies to NOX1 (Mox1, H75, and sc-25545), NOX4 (H-300, sc-30141), COX-2 (29, sc-19994), and TGF*β*1/2 (12Y-1, sc-80346L) were obtained from Santa-Cruz Biotechnology (Dallas, TX, USA). Hybond-ECL membrane was supplied by GE Healthcare; enzymes were from Thermo Scientific (Rockford, IL, USA). qPCRmix-HS and qPCRmix-HS SYBR + ROX master mixes were purchased from Evrogen (Moscow, Russia). The unmodified DNA oligonucleotides were supplied by Litech (Moscow, Russia), the Taqman probes were synthesized by Syntol (Moscow, Russia), and the RNA oligonucleotides were obtained from DNA Synthesis Ltd. (Moscow, Russia). Huh7 cells were a kind gift of Professor R. Bartenschlager (Heidelberg University, Germany).

### 2.2. Plasmid Construction

C-myc tagged expression plasmids for full-length NS5A protein and N- and C-terminal fragments were constructed based on the pCMV-Tag3 vector (Agilent, Santa-Clara, CA, USA). A full-length NS5A (1–447 aa) fragment encoding a 1b genotype NS5A sequence (AJ238799) was amplified using oligonucleotides P1 and P2 (Table 1). The product was digested with* Pst*I and* Hind*III endonucleases and cloned into the respective sites of pCMV-Tag3. NS5A fragments corresponding to domains I (DI, 1–249 aa), II (D2, 250–355 aa), and III (D3, 356–447 aa) were constructed in a similar fashion using pairs of oligos P1 and P3, P4 and P5, and P2 and P6 (Table 1), respectively. All plasmids were confirmed by sequencing.

### 2.3. Cell Culture and Transfection

The human hepatoma Huh7 cell line was maintained and transfected as described in [8]. For RNA interference, siRNAs (see Table 2) were annealed in a buffer (5 *μ*M each siRNA in 5 mM Tris-HCl, pH 7.5, 1 mM EDTA) by heating at 65°C for 5 min and then slow cooling to room temperature. Transfection of the cells with the obtained siRNA duplexes was performed using Lipofectamine 2000 according the manufacturer's specification using 100 pmol of each duplex per well of 24-well plate or 400 pmol per well of 6-well plate. To prevent expression of TGF*β*1-dependent genes, the NS5A-expressing cells were treated with anti-TGF*β*1/2 neutralizing antibodies 18 h after transfection and subjected to analysis after additional 30 h. Alternatively, anti-HCV core antibodies were added as a negative control. In addition, in some experiments, the cells were treated with 100 *μ*M 4-methylpyrazole (4-MP), 100 *μ*M BAPTA-AM, or 100 *μ*M Ru360.

### 2.4. Measurement of Reactive Oxygen Species

ROS levels in cells grown in 24-well plates were measured using two low molecular weight dyes: 2′,7′-dichlorodihydrofluorescein diacetate (H_2_DCFDA) and dihydroethidium (DHE), whose fluorescence is dependent on levels of H_2_O_2_ and O_2_^∙−^, respectively. In case of both dyes the cell culture medium was removed 28 h after transfection and replaced with the medium containing 25 *μ*M H_2_DCFDA or DHE. After incubation at room temperature for 30 min the media was removed, and the cells were washed 10 times with PBS (500 *μ*L/well). The fluorescence intensities (FLI) were measured in PBS (200 *μ*L/well) using Plate CHAMELEON V reader (Hidex Ltd.) with excitation at 485 nm and emission at 535 nm in case of H_2_DCFDA or with excitation at 510 nm and emission at 590 nm in case of DHE.

### 2.5. Western Blotting

Western blotting was performed as described previously [8]. Primary antibodies were used at the following concentrations: anti-*β*-actin: 0.2 *μ*g/mL; anti-NOX1: 0.2 *μ*g/mL; anti-NOX4: 0.4 *μ*g/mL; anti-COX-2: 0.4 *μ*g/mL; and anti-CYP2E1: 0.5 *μ*g/mL. The HRP-conjugated anti-mouse and anti-rabbit secondary antibodies were used at concentrations of 0.2 *μ*g/mL. The HRP-conjugated antibodies to the c-myc tag were used at 0.2 *μ*g/mL concentration.

### 2.6. Quantitative Real-Time PCR (qRT-PCR)

Total RNA was purified and cDNA was synthesized as described previously [8]. Levels of human NOX1, NOX4, COX-2, TGF*β*1, CYP2E1, Ero1*α*, and Ero1*β* transcripts were quantified according to SYBR Green approach using qPCRmix-HS SYBR + ROX mixture and the primers listed in Table 3. Levels of *β*-actin transcript as the loading control were determined using previously described Taqman probes [8]. Thermal cycling conditions for both types of real-time PCR included activation at 55°C (5 min) and 95°C (10 min) followed by 40 cycles each of denaturation at 95°C (10 s) and annealing/elongation at 57°C (1 min).

### 2.7. Quantification of TGF*β*1 in Culture Medium by ELISA

Huh7 cells were grown on 24-well plates and transfected as described above. Forty-eight h after transfection the cell culture medium was collected, cell debris was removed by centrifugation (3,000 rpm, 10 min), and concentrations of TGF*β*1 were quantified by Human TGF-beta1 Platinum ELISA kit (eBioscience, San Diego, CA, USA) according to manufacturer's specification.

### 2.8. Statistical Analysis

Statistical analysis was performed using BioStat 2009 software (AnalystSoft, Vancouver, Canada). The results are presented as means ± SD. Shapiro-Wilk *W* test was used to confirm normal distribution of the data. Homogeneity of the variance was studied by Levene's test using SPC for Excel Software (BPI Consulting, LLC, Cypress, TX, USA). Significant differences were determined using one-way ANOVA followed with a Tukey-Kremer* post hoc* analysis. Statistical differences between treated and untreated cells in experiments with neutralizing antibodies were determined using a paired Student's *t*-test.

## 3. Results

### 3.1. Domain I of NS5A Protein Induces Oxidative Stress

**Figure 1:**
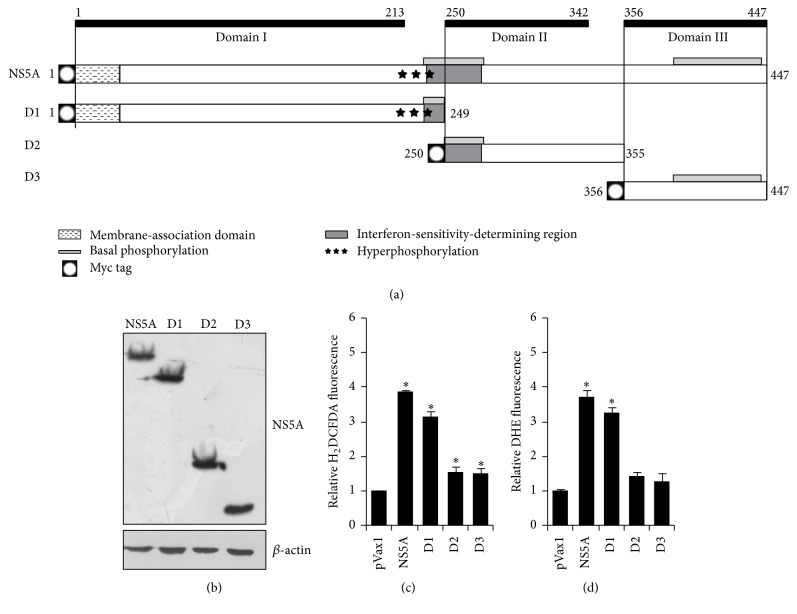
Domain I of HCV NS5A protein is responsible for induction of oxidative stress in Huh7 cells. (a) Schematic representation of truncated forms of NS5A used in the study. Note that all NS5A expression constructs contained an N-terminal c-*myc* tag. The obtained plasmids were transfected into Huh7 cells, which were subjected to detection of NS5A by Western-blot 48 h after transfection using an anti-*myc* antibody (b) or to quantification of ROS levels in H_2_DCFDA (c) or DHE (d) assays as described in Section 2. (c and d) Results are presented as mean ± SD from seven replicates. ^*∗*^ *P* < 0.001* versus* pVax1 (ANOVA with Tukey-Kramer* post hoc* analysis).

The expression levels of full-length (1–447 aa) NS5A or its individual domains 1 (1–249 aa), 2 (250–355 aa), and 3 (356–447 aa) (Figure 1(a)) were verified by western blotting using an antibody recognizing an N-terminal c-myc tag, displayed by all NS5A proteins, upon transfection of Huh7 cells (Figure 1(b)). Full-length as well as fragments of NS5A were expressed in Huh7 cells to comparable levels.

The ability of individual domains of NS5A protein to induce oxidative stress was investigated with two low molecular weight dyes, namely, 2′,7′-dichlorodihydrofluorescein diacetate (H_2_DCFDA) and dihydroethidium (DHE). H_2_DCFDA is absorbed by cells and then converted into its deesterified form H_2_DCF, which is oxidized into a fluorescent product in an H_2_O_2_-dependent fashion [16]. Treatment of Huh7 cells transfected with NS5A-expressing plasmids revealed that the full-length protein as well as domain I (D1) induced ROS production (Figure 1(c)). In contrast, domains II and III only slightly affected the level of fluorescence compared to cells transfected with the empty control vector pVax1 (Figure 1(c)). The same results were obtained with DHE, a superoxide anion-specific dye [16]. Again, a pronounced increase in production levels of superoxide anion was observed only for the full-length NS5A and its domain I (Figure 1(d)). Thus NS5A as well as D1 induce ROS.

### 3.2. Induction of Oxidative Stress by NS5A Protein Is Partially Mediated by NOX1, NOX4, and Cyclooxygenase 2

**Figure 2:**
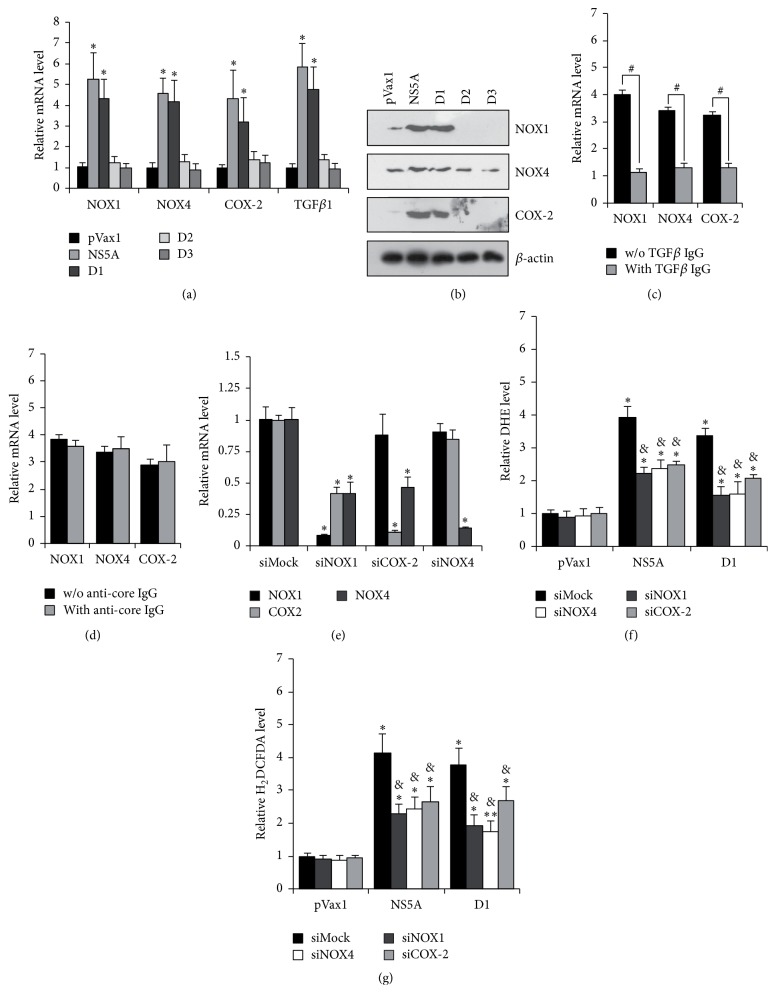
Domain I of NS5A protein triggers ROS production* via* activation of the TGF*β*1 → NOX1 → COX-2 → NOX4 cascade. (a, b) Huh7 cells were transfected with NS5A-expression constructs and subjected to gene expression analysis by real-time RT-PCR (a) or Western blotting (b). (c, d) NS5A-expressing cells were treated with anti-TGF*β*1/2 antibodies (c) or anti-HCV core antibodies as negative control (d), and after an additional 30 hrs gene expression was quantified using RT-qPCR. (d–f) Huh7 cells expressing full-length NS5A were transfected with siRNA and 48 hrs later subjected to gene expression analysis by RT-qPCR (e) or measurement of ROS levels using the DHE (f) or H_2_DCFDA (g) assays. Results are mean ± SD from six replicates; ^*∗*^ *P* < 0.01 or ^*∗∗*^ *P* < 0.05 versus DMSO-treated cells transfected with pVax1 (ANOVA with Tukey-Kramer* post hoc* analysis); ^&^ *P* < 0.01 versus DMSO-treated cells transfected with the respective NS5A form (ANOVA with Tukey-Kramer* post hoc* analysis); ^#^ *P* < 0.01 (*t*-test).

It has previously been reported that HCV infection provokes oxidative stress by inducing expression of NOX1 and NOX4, and in case of NOX4 the effect has been shown to be mediated by both structural and nonstructural proteins of the virus [12, 13]. Here, the role of NS5A on expression of NOX1 and NOX4 was studied by real-time RT-PCR and Western-blot analysis. As shown in Figures 2(a) and 2(b), the full-length NS5A protein and domain 1 induce expression of NOX1 and, to a lesser extent, of NOX4 as well as of TGF*β*1 and cyclooxygenase 2 (COX-2) (Figures 2(a) and 2(b)). Induction of TGF*β*1 production was verified by ELISA (Table 4). These two latter proteins have previously been implicated in the regulation of NOX1 and NOX4 in response to inflammatory stimuli [17]. The role of TGF*β*1 and COX-2 in NS5A induced induction of NOX1 and NOX4 was verified using neutralizing antibodies and RNA interference approaches. Treatment of NS5A-expressing cells with anti-TGF*β*1/2 antibodies prevented activation of both NOX1 and NOX4, as was revealed by RT-qPCR (Figure 2(c)). At the same time, anti-COX-2 siRNAs suppressed expression not only of cyclooxygenase 2 but also of NOX4 (Figure 2(d)). In addition, anti-NOX1 siRNA caused a decrease of NOX1, NOX4, and COX-2 transcripts (Figure 2(d)) supporting the existence of a TGF*β*1 → COX1 → COX-2 → NOX4 cascade.

Next, the same approaches were employed to reveal the role of these proteins in NS5A-induced oxidative stress. Indeed, anti-NOX1, NOX4, and COX-2 siRNAs decreased ROS production by 1.6–1.8-fold, as was revealed in DHE and H_2_DCFDA assays (Figures 2(e) and 2(f)) but did not eliminate it down to background levels. Thus, NADPH oxidases 1 and 4 represent an important, but not the only, source of ROS, in HCV NS5A expressing cells.

### 3.3. CYP2E1 Is Another Source of ROS in NS5A-Expressing Cells

**Figure 3:**
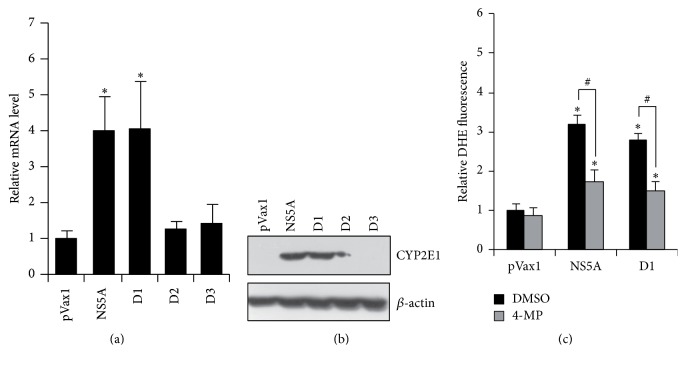
HCV NS5A protein induces expression of cytochrome P450 2E1 which contributes to ROS production. Huh7 cells were transfected with NS5A-expressing constructs and subjected to quantification of CYP 2E1 levels by real-time RT-PCR (a) or Western blotting (b) 40 hrs after transfection. (c) 18 hrs after transfection NS5A expressing Huh7 cells were treated with 100 *μ*M 4-methylpyrazole (4MP) for additional 12 hrs prior to quantification of ROS levels in DHE assay. Results are mean ± SD from six replicates; ^*∗*^ *P* < 0.01 versus DMSO-treated cells transfected with pVax1; ^#^ *P* < 0.01 (ANOVA with Tukey-Kramer* post hoc* analysis).

Cytochrome P450 2E1 is considered as a significant source of ROS [5]. Using real-time RT-PCR and Western-blot analysis it was revealed that NS5A and its domain 1 cause a pronounced increase in CYP2E1 expression (Figures 3(a) and 3(b)). In contrast, domains 2 and 3 again had no detectable effect on cytochrome expression. Treatment of NS5A-expressing cells with 4-methylpyrazole, a low molecular weight inhibitor of CYP2E1 enzymatic activity, suppressed production of superoxide anion by app. 40% (Figure 3(c)). Thus, CYP2E1 represents a novel source of ROS which is induced by HCV NS5A protein.

### 3.4. HCV NS5A Protein Does Not Affect Expression of ER Oxidoreductins 1*α* and 1*β*

**Figure 4:**
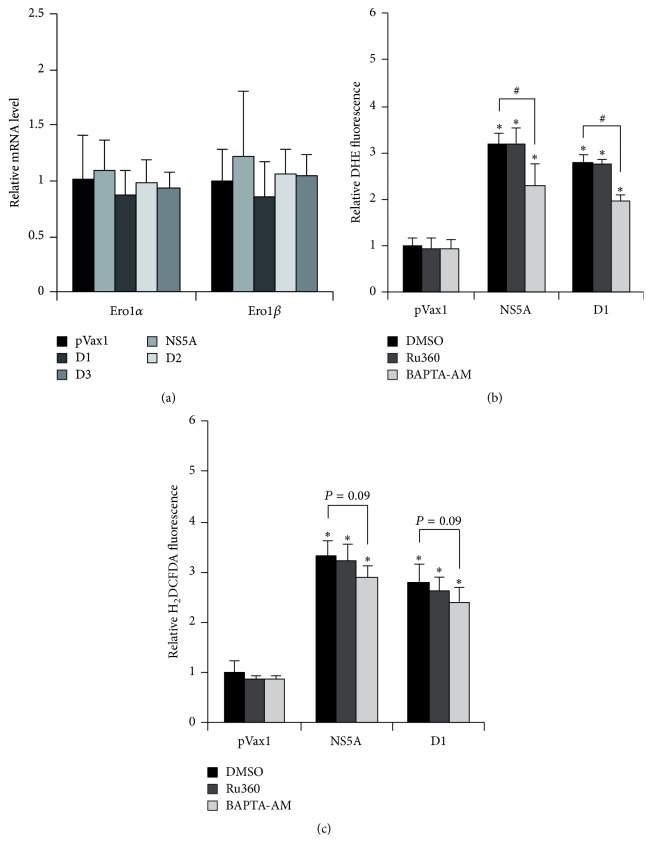
ER oxidoreductins 1*α* and 1*β* and calcium signaling have no significant role in induction of oxidative stress by NS5A protein. (a) Huh7 cells were transfected with NS5A-expressing plasmids, and levels of Ero1*α* and 1*β* transcripts were analyzed 40 hrs after transfection by real-time RT-PCR. (b, c) 18 hrs after transfection NS5A expressing Huh7 cells were treated with calcium chelator BAPTA-AM or inhibitor or mitochondria Ca^2+^ uniporter Ru360, and ROS levels were analyzed 28 hrs after transfection in DHE (b) or H_2_DCFDA (c) assays as described in Section 2. Results are mean ± SD from six replicates; ^*∗*^ *P* < 0.01 versus DMSO-treated cells transfected with pVax1; ^#^ *P* < 0.01 between the indicated groups (ANOVA with Tukey-Kramer* post hoc* analysis).

Another probable group of enzymes involved in induction of oxidative stress are the ER oxidoreductins 1*α* and 1*β* (Ero1*α* and Ero1*β*) which produce H_2_O_2_ and regulate redistribution of calcium ions between ER and mitochondria [18]. Previously we found that HCV core induces Ero1*α* at transcriptional level [14]. To unveil a role for both enzymes in oxidative stress, their expression in naive and NS5A-expressing Huh7 cells was measured by real-time RT-PCR. However, neither full-length NS5A nor its individual domains affected the transcript levels of these enzymes (Figure 4(a)).

### 3.5. Efflux of Calcium Ions from ER Does Not Contribute Significantly to NS5A-Induced Oxidative Stress

Next, the role of calcium ions in NS5A-mediated induction of oxidative stress was estimated. It was performed using two compounds: BAPTA-AM, a general cell-permeable Ca^2+^ chelator, and Ru360, an inhibitor of calcium uniporter which mediates influx of the ions from the ER directly into mitochondria. Cytoplasmic calcium chelator BAPTA-AM caused only 30% reduction in ROS production in DHE assays (Figure 4(b)). In H_2_DCFDA assays a similar reduction was observed (Figure 4(c)). Ru360 did not affect ROS production at all. Thus, calcium signaling has only minor input in NS5A-triggered oxidative stress.

## 4. Discussion

Previously it has been reported that one of the key mechanisms by which HCV core protein induces oxidative stress is the efflux of calcium ions from the ER and their accumulation in mitochondria ([2, 5] and references herein). In the case of NS5A, release of Ca^2+^ from ER stores has also been reported, although this release has been considered to be a consequence of oxidative stress [19]. Our data support these observations, as calcium chelator BAPTA-AM had only a minor effect, whereas Ru360, an inhibitor of direct flux of Ca^2+^ from ER to mitochondria, had no effect at all on NS5A-mediated ROS production. Therefore, HCV core and NS5A proteins are likely to induce oxidative stress by different mechanisms.

The other reported mechanism of HCV-induced ROS production involves induction of NADPH oxidases 1 and 4 [12, 13]. In these studies, the ability to trigger NOX1 expression was assigned to the core protein [12], whereas enhanced expression of NOX4 was observed in case of both structural and nonstructural viral proteins [13]. Our data suggest that expression of both NOX genes can be mediated by NS5A protein, and their induction is achieved through activation of TGF*β*1 expression. In addition, we observed that both NOX1 and NOX4 can contribute to superoxide anion production using an siRNA approach, in contrast to data in the literature, which suggest that* in vitro* production of ROS by recombinant NOX4 is limited to H_2_O_2_ [20]. However, production of H_2_O_2_ has been described mainly for the recombinant NOX4 [20], whereas various independent groups have also reported decreased superoxide production in cells with downregulated NOX4 [12, 21].

In 2011 Sancho et al. revealed that induction of NADPH oxidases in response to proinflammatory stimuli was regulated by the cascade TGF*β*  → NOX1 → COX-2 → NOX4 in Chang liver CCL-13 cells [17]. We confirmed the existence of this cascade in NS5A expressing Huh7 cells using anti-TGF*β*1/2 antibodies and anti-NOX and COX2 siRNAs; however, we found no evidence for an activation of this cascade in HCV core expressing Huh7 cells [14]. Interestingly, TGF*β* induction was earlier reported to depend on calcium signaling, at least in the replicative HCVcc infectious system [22]. However, using the Ca^2+^ chelator BAPTA-AM, we noticed only minor effects on TGF*β* induced oxidative stress.

Cytochrome P450 2E1 is a ROS-producing enzyme which is involved in catabolism of an array of endogenous and exogenous compounds including ethanol [23]. It is expressed mainly in the liver and is localized mostly in the ER membrane [23], where HCV replication occurs [1]. Here CYP2E1 was identified as an important source of ROS in NS5A expressing cells. Previously it has been shown that cooverexpression of CYP2E1 with HCV core protein activates ROS production in response to xenobiotic treatment much more potently than any of these proteins expressed alone [24]. Elevated CYP2E1 expression was also described in liver of chronic hepatitis C patients with mild fibrosis [25]. Thus our data present the first evidence that HCV NS5A can directly induce CYP2E1 expression which in turn contributes to oxidative stress.

The ER represents an organelle which produces significant amount of ROS mostly by the protein folding machinery [5]. ER oxidoreductins 1*α* and 1*β* are implicated in disulphide bond formation, with H_2_O_2_ being a major by-product [26]. In addition, it has recently been discovered that Ero1*α* is also involved in a control of Ca^2+^ translocation from ER directly to mitochondria through mitochondria-associated membranes (MAM) [18]. To date there is no literature on regulation of Ero1*α* or *β* by viral infections, except our recent report for the case of HCV core protein. The only two pieces of evidence that have been reported are the suppression of Ero1 expression in epithelial cells from HIV-infected individuals on highly active antiretroviral therapy [27] and our recent data on induction of Ero1*α* by HCV core which contributes to the oxidative stress [14]. Here in the case of HCV, NS5A has no effect on Ero1*α* or *β* expression. Thus, HCV core and NS5A proteins exhibit different effect of Ero1*α* expression and on consequent calcium signaling.

NS5A represents a 447 aa polyprotein containing three distinct domains: 1 (aa 1–213), 2 (250–342), and 3 (356–447) (Figure 1(a)) [28]. The present study was based on NS5A fragments containing one of these domains: aa 1–249, 250–355, and 356–447 (Figure 1(a)). Our data suggest that domain 1 (D1) contains a strongly prooxidant activity. Previously it was shown that this zinc-binding domain has a distinct conformation and can form homodimers [29], whereas domains 2 and 3 are unfolded [30, 31]. However, the ability of D1 to enhance ROS production may also be due to its localization to the ER; indeed, in D1, the first 30 N-terminal aa of NS5A form a hydrophobic membrane-associating *α*-helix [32]. The phosphorylation status of NS5A is unlikely to contribute to the generation of oxidative stress, because all phosphorylation sites are localized within D3. Furthermore, hyperphosphorylation is known to require coexpression of other nonstructural viral proteins, which were absent in our study [1, 28]. Finally, the prooxidant activity of NS5A is unlikely to be associated with ability of the protein to ensure HCV escape from interferon *α*, as the interferon-sensitivity-determining region (ISDR) is localized between D1 and D2 [1].

Altogether, our data show that HCV-induced oxidative stress can be mediated not only by altered calcium signaling and mitochondrial dysfunctions, but also by overexpression of nonmitochondrial proteins including NADPH oxidases 1 and 4, cyclooxygenase 2, and cytochrome P450 2E1. Therefore, special attention should be paid to the investigation of the role of these proteins in viral pathogenesis as well as in nonvirally induced pathologies that are characterized by oxidative stress. Currently both NOX1 and NOX4 and CYP2E1 are implicated in development of inflammation and liver fibrosis [4, 33–35], whereas pharmacological inhibitors of these enzymes have been shown* in vivo* to prevent hepatocyte death and attenuate fibrosis progression [36, 37]. CYP2E1 can also contribute to aggravated progression of liver disease in CHC patients addicted to heavy alcohol consumption [3]. Apart from virus pathogenicity, certain attention should be given in the future to dissecting role of various sources of ROS in virus life cycle. It has been shown that exogenous H_2_O_2_ affects virus replication; however no attempts have been reported to estimate the impact of endogenous ROS production and its sources/localization on replication capacity. We have recently demonstrated that ROS can affect infectivity of the newly formed virions, which is prevented by HCV-triggered induction of glutathione peroxidase 4 (GPx4) [38]. But again, no data exist in literature on possible role of cellular ROS-producing enzymes on early or late stages of virus propagation. Last but not least, detailed investigation of the mechanisms by which viral proteins induce oxidative stress can be used to develop effective DNA vaccines. Indeed, previously, we showed for HIV reverse transcriptase that the prooxidant activity of its various forms correlated with its ability to induce specific interferon *γ* response thus linking redox alterations and immunogenic properties of virus proteins [39].

In conclusion, we identified mechanisms of oxidative stress induction by HCV NS5A protein.

## Figures and Tables

**Table 1:** Oligonucleotides used for plasmid construction.

Oligonucleotide	Restriction site	Sequence
P1	*Pst*I	5′-ATTCTGCAGTCCGGCTCGTGGCTAAGA-3′
P2	*Hind*III	5′-AATAAGCTTTTAGCAGCAGACGACGTCCTC-3′
P3	*Hind*III	5′-ATTAAGCTTTTAGGAGTCATGACGGGTAGTG-3′
P4	*Pst*I	5′-ATTCTGCAGCCGGACGCTGACCTCATC-3′
P5	*Hind*III	5′-ATTAAGCTTTTATGGAGGTGGTATCGGAGG-3′
P6	*Pst*I	5′-AATCTGCAGCGGAGGAAGAGGACGGTT-3′

**Table 2:** Oligonucleotides used to assemble short interfering RNA (siRNA).

Target transcript	Abbreviation	Sequence
No	siMock	5′-GUAAGACACGACUUAUCGCdTdT-3′
		5′-GCGAUAAGUCGUGUCUUACdTdT-3′
NOX1	siNOX1	5′-UCAUAUCAUUGCACAUCUAdTdT-3′
		5′-UAGAUGUGCAAUGAUAUGAdTdT-3′
NOX4	siNOX4	5′-GCCUCUACAUAUGCAAUAAdTdT-3′
		5′-UUAUUGCAUAUGUAGAGGCdTdT-3′
COX-2	siCOX2	5′-UGAAAGGACUUAUGGGUAAdTdT-3′
		5′-UUACCCAUAAGUCCUUUCAdTdT-3′

**Table 3:** Primers used for quantification of gene transcription levels by real-time RT-PCR.

Transcript		Sequence
NOX1	Sense	5′-TTAACAGCACGCTGATCCTG-3′
	Antisense	5′-CTGGAGAGAATGGAGGCAAG-3
NOX4	Sense	5′-GCTGACGTTGCATGTTTCAG-3′
	Antisense	5′-CGGGAGGGTGGGTATCTAA-3′
COX-2	Sense	5′-CCATGTCAAAACCGAGGTGTAT-3′
	Antisense	5′-TCCGGTGTTGAGCAGTTTTCT-3′
TGF*β*1	Sense	5′-GCAGCACGTGGAGCTGTA-3′
	Antisense	5′-CAGCCGGTTGCTGAGGTA-3′
CYP 2E1	Sense	5′-GACTGTGGCCGACCTGTT-3′
	Antisense	5′-ACTACGACTGTGCCCTTGG-3′
Ero1*α*	Sense	5′-GCATTGAAGAAGGTGAGCAA-3′
	Antisense	5′-ATCATGCTTGGTCCACTGAA-3′
Ero1*β*	Sense	5′-GGGCCAAGTCATTAAAGGAA-3′
	Antisense	5′-TTTATCGCACCCAACACAGT-3′

**Table 4:** Secretion of TGF*β*1 from Huh7 cells transfected with pVax1 or plasmids expressing full-length NS5A or its domain 1.

	pVax1	NS5A	D1
TGF*β*1 (pg/mL)	<375^**∗**^	690 ± 137	565 ± 80

^**∗**^The value was below sensitivity of kit for TGF*β*1.

## Abbreviations

HCV: Hepatitis C virus
CHC: Chronic hepatitis C
NOX: NADPH oxidase
CYP2E1: Cytochrome P450 2E1
Ero1*α*: ER oxidoreductin 1*α*
TGF*β*1: Transforming growth factor *β*1.
